# Validation of the mothers object relations scales in 2–4 year old children and comparison with the child–parent relationship scale

**DOI:** 10.1186/1477-7525-11-49

**Published:** 2013-03-21

**Authors:** Douglas E Simkiss, Fiona MacCallum, Emma EY Fan, John M Oates, Peter K Kimani, Sarah Stewart-Brown

**Affiliations:** 1 Warwick MedicalSchool, University of Warwick, Coventry, UK; 2 Department of Psychology, University of Warwick, Coventry, UK; 3 Child and Youth Studies Group, Open University, Milton Keynes, UK

**Keywords:** Parent, Child, Relationship, Outcome measure, Psychometrics, Validity, Internal consistency

## Abstract

**Background:**

The quality of the parent–child relationship has an important effect on a wide range of child outcomes. The evaluation of interventions to promote healthy parenting and family relationships is dependent on outcome measures which can quantify the quality of parent–child relationships. Between the Mothers’ Object Relations – Short Form (MORS-SF) scale for babies and the Child–parent Relationship Scale (C-PRS) there is an age gap where no validated scales are available. We report the development and testing of an adaptation of the MORS-SF; the MORS (Child) scale and its use in children from the age of 2 years to 4 years. This scale aims to capture the nature of the parent–child relationship in a form which is short enough to be used in population surveys and intervention evaluations.

**Methods:**

Construct and criterion validity, item salience and internal consistency were assessed in a sample of 166 parents of children aged 2–4 years old and compared with that of the C-PRS. The performance of the MORS (Child) as part of a composite measure with the HOME inventory was compared with that of the C-PRS using data collected in a randomised controlled trial and the national evaluation of Sure Start.

**Results:**

MORS (Child) performed well in children aged 2–4 with high construct and criterion validity, item salience and internal consistency. One item in the C-PRS failed to load on either subscale and parents found this scale slightly more difficult to complete than the MORS (Child). The two measures performed very similarly in a factor analysis with the HOME inventory producing almost identical loadings.

**Conclusions:**

Adapting the MORS-SF for children aged 2–4 years old produces a scale to assess parent–child relationships that is easy to use and outperforms the more commonly used C-PRS in several respects.

## Background

The quality of the parent–child relationship has an important effect on a wide range of child outcomes, including mental health throughout the life course [1-5]; healthy lifestyles [6], smoking and alcohol use [7], teenage pregnancy [8], injuries [9], physical health [3,10-13], social skills [5,14] and educational attainment [15,16].

**Figure 1:**
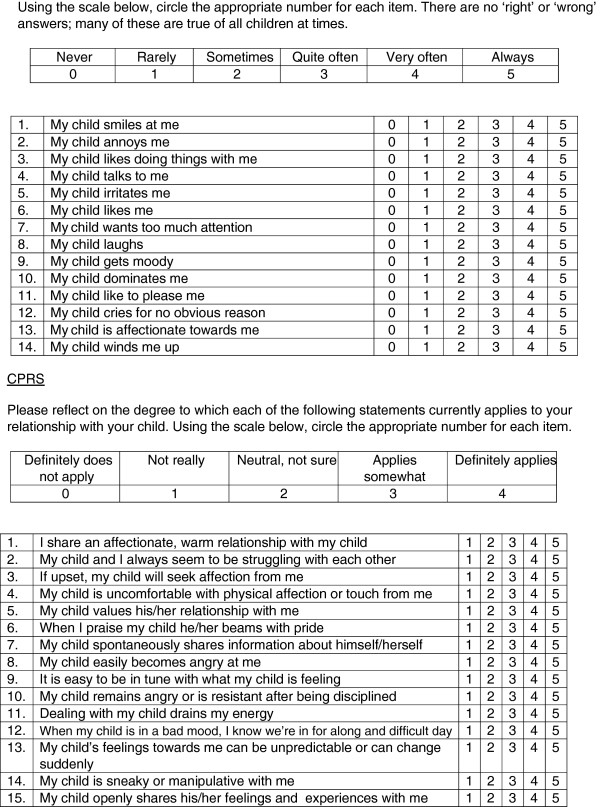
MORS (Child) & CPRS scales.

The evaluation of interventions to promote healthy parenting and family relationships is dependent on outcome measures which can quantify the quality of parent–child relationships. The Child–parent Relationship Scale (C-PRS) (Figure 1) [17] is validated for this purpose for 3 year olds and older [18] and has been used in the National Head Start Impact Study [19] in the US and the National Evaluation of Sure Start [20] in the UK. The internal and external validity of the Mothers’ Object Relations Scale – Short Form (MORS-SF) and its utility in clinical and research settings have been established for infants in studies in England and Hungary [21-24], but there are no scales suitable for use with the age range two to four years.

We report here on the development and testing of an adaptation of the MORS-SF, the MORS (Child) scale, and its use in children from the age of 2 years to 4 years (Figure 1). The MORS (Child) incorporates all the items from the MORS-SF scale changing the word ‘baby’ in each question to ‘child’. This scale aims to capture the nature of the parent–child relationship in a form which is short enough to be used in population surveys and intervention evaluations. It is a fourteen item scale developed as a screening tool to identify potential problems in the early mother-infant relationship; particularly in a mother’s working model of attachment. The scale taps into mothers’ psychodynamic processes but the questions describe aspects of infant behaviour, so limiting the social desirability response bias [22]. The descriptors were derived from research examining mothers’ narrative accounts of their perceptions of infants’ feelings, cognitions and behaviours [25] and measure two orthogonal factors; a mother’s perceptions of her infant’s emotional ‘warmth’ and perceived ‘invasiveness’ towards herself. The MORS-SF axis items have been shown to possess stable and internally coherent scales in the infant (6–52 weeks of age) population [24].

## Methods

### Validation of MORS (Child)

#### Participants and data collection

Quantitative data were collected from 166 parents of children aged 2–4 years attending 5 children’s centres and nurseries in Warwickshire. A sample of 150 parents provided sufficient power to investigate the psychometric properties of the questionnaires. Parents were asked to complete the MORS (Child) and the C-PRS scales. This took less than 10 minutes. Participants were given the option of completing the scales on the spot as they attended the children’s centre, or in their own time. For use in primary care and clinical settings, ease of administration and completion, and brevity in numbers of items are important considerations. Participants were asked to rate the ease of completion of each item in each scale quantitatively and invited to add comments in text.

#### Item salience

The frequencies of complete responses to MORS (Child) and C-PRS were examined to assess the perceived relevance and adequacy of MORS (Child) to the target population in Warwickshire. To assess the relevance, sensitivity and signs of inappropriateness, the incidence of missing item responses was considered. In addition, the distributions of responses from complete responders highlighted the frequency of population responses.

#### Construct validity

Exploratory factor analysis was undertaken using SPSS statistical software to identify the factors assessed by the MORS (Child) and the C-PRS in this population. Extracted factors were then compared to those measured by the MORS-SF, and the C-PRS with older children.

#### Internal consistency

Cronbach’s alpha was calculated for all subscales at ages 2, 3 and 4 years. Internal consistency estimates of >0.70 were sought [26].

#### Criterion validity

Scale and item scores were examined for floor and ceiling effects and the normality assumption investigated using the Shapiro-Wilk test. Correlations between scores on MORS (Child) and the C-PRS were calculated using Spearman’s rank correlation coefficients.

#### External validation

The performance of the MORS (Child) scale was assessed in conjunction with the HOME inventory [27] in factor analysis using data collected on 287 families of children age 2-4yrs in a randomised controlled trial in south Wales [28]. Results were compared with the performance of the C-PRS in a similar published analysis undertaken in the national evaluation of Sure Start using 120 families in each of 150 randomly sampled Sure Start areas [20]. Invasiveness scores in the MORS (Child) scale were rescaled from 0–35 to 6–30 to correspond to the C-PRS conflict subscale. Similarly, we rescaled MORS (Child) warmth scores from 0–35 to 9–45 as in C-PRS closeness subscale.

#### Ethics

This study was given a favourable opinion by the Biomedical Research Ethics Sub-Committee of Warwick Medical School. Written consent for publication was obtained from participants.

## Results

### Participants and response rates

166 parents (113 female, 14 male and 39 gender not given) completed the MORS (Child) and C-PRS scales: 57 parents of children aged 4 years old, 50 of children aged 3 years and 59 of children aged 2 years. For the MORS (child) scale 110 parents found it easy to complete, 17 quite easy, 35 okay, none quite difficult and one parent said it was difficult to complete (n=163). For the C-PRS scale 104 parents reported it was easy, 22 quite easy, 37 okay, 2 quite difficult and none found it difficult to complete (n=165).

Comments on the ease of completion of the MORS (child) were:

‘*My child has a lot of good and angry feelings at home’*

‘As the questions asked ‘my child’…, if the question was my child's behaviour…, I may have responded differently. I thought question 7 was difficult as I like to give my child lots of attention so how to measure too much was not easy’

‘Q 14 doesn't really apply in the context of children’

‘Good but there seems to be no questions about the parent's mood, only the child's’

‘I found the questions bit hard to understand. e.g. my child annoys me - it depends what I’m trying to do at the time!’

‘All questions ok except question 4 as child isn't talking yet’

Comments on the ease of completion of the C-PRS were:

‘Just concentrating on filling this in whilst watching my son’

‘My child often wants to go to his dad when he has hurt himself’

‘Quite difficult as some days are good and some days aren't, or can be more difficult depending on how I am feeling. However, we do have a close relationship and a good routine. Some days go better than others’

‘The scale definitely apply etc., could be re-worded to be simpler’

‘Qs 5&15 are unsure as my child is quite young and doesn't really share her feelings as such’

‘Some of the questions were a bit too general. Q11: child drains energy - does this mean emotional or physical?’

‘Easy, however every other line should be shaded to make it even easier’

#### Item salience

**Figure 2:**
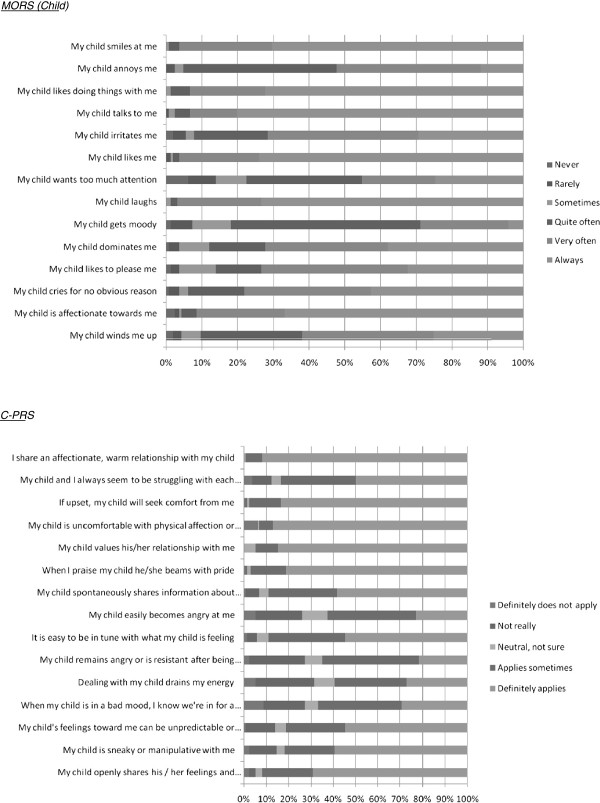
Questionnaire responses.

All parents completed all items in both scales. Three parents did not answer the question asking about ease of completion of the MORS (Child) questionnaire and one parent did not complete this for the C-PRS. Figure 2 shows the item response frequencies for both scales. Responses to the C-PRS show more evidence of skewed distribution than those for the MORS (Child). Some response categories were unused on some items in both scales.

#### Construct validity

A principal components analysis with orthogonal rotation (varimax) was conducted on the 14 MORS (Child) items. The Kaiser-Meyer-Olkin measure of sampling adequacy was .794 (above recommended value of .6), and Bartlett’s test of sphericity was significant (χ^2^(91) = 729.55, *p*<.001), showing factorability of the items. Furthermore, the communalities were all above .3, confirming that each item shared some common variance with other items. Two factors had eigenvalues >1 and together explained 45% of the variance. All items in the MORS (Child) questionnaire mapped onto one or other of these two factors. These matched the constructs described for the MORS-SF labelled ‘warmth’ and ‘invasiveness’ (Table 1).

**Table 1:** Rotated component matrix for MORS (Child)

	**Component**	
**Item**	**1**	**2**
7. My child wants too much attention	**0.774**	0.159
14. My child winds me up	**0.745**	−0.023
5. My child irritates me	**0.734**	0.137
10. My child dominates me	**0.712**	0.165
2. My child annoys me	**0.661**	−0.022
9. My child gets moody	**0.594**	0.230
12. My child cries for no obvious reason	**0.475**	0.278
1. My child smiles at me	0.038	**0.726**
3. My child likes doing things with me	0.110	**0.689**
8. My child laughs	0.098	**0.656**
6. My child likes me	0.109	**0.641**
13. My child is affectionate towards me	0.065	**0.601**
4. My child talks to me	0.238	**0.555**
11. My child like to please me	0.110	**0.546**

Extraction Method: Principal Component Analysis.

Rotation Method: Varimax with Kaiser Normalization.

Rotation converged in 3 iterations.

A principal components analysis with orthogonal rotation (varimax) was conducted on the 15 C-PRS items. Factorability of items was shown by the Kaiser-Meyer-Olkin measure of sampling adequacy value of .796, the significance of Bartlett’s test of sphericity (χ^2^(105) =658.39, *p*<.001), and the fact that the communalities were all above .3 (except for item 4). Two factors had eigenvalues >1 and together explained 42% of the variance. With the exception of item 4 ‘*my child is uncomfortable with physical affection or touch from me*’, all items mapped onto one of these two factors. The item composition of these matched the subscales previously described [17] for C-PRS labelled ‘closeness’ and ‘conflict’, Table 2. In previous analyses with children aged 3–7 years, item 4 loaded on component 1; ‘conflict’.

**Table 2:** Rotated component matrix for C-PRS

	**Component**	
**Item**	**1**	**2**
8. My child easily becomes angry at me	**0.717**	−0.075
11. Dealing with my child drains my energy	**0.706**	0.107
12. When my child is in a bad mood, I know we’re in for a long and difficult day	**0.694**	0.259
10. My child remains angry or is resistant after being disciplined	**0.689**	0.018
13. My child’s feelings towards me can be unpredictable or can change suddenly	**0.651**	0.198
14. My child is sneaky or manipulative with me	**0.637**	0.267
2. My child and I always seem to be struggling with each other	**0.473**	0.355
6. When I praise my child he/her beams with pride	0.097	**0.649**
7. My child spontaneously shares information about himself/herself	0.270	**0.623**
3. If upset, my child will seek affection from me	−0.143	**0.617**
15. My child openly shares his/her feelings and experiences with me	0.153	**0.614**
1. I share an affectionate, warm relationship with my child	0.275	**0.592**
9. It is easy to be in tune with what my child is feeling	0.014	**0.579**
5. My child values his/her relationship with me	0.201	**0.574**
4. My child is uncomfortable with physical affection or touch from me	0.116	0.320

Extraction Method: Principal Component Analysis.

Rotation Method: Varimax with Kaiser Normalization.

Rotation converged in 3 iterations.

We have evaluated the conflict subscale of the C-PRS as recommended for 3 year olds plus, and as suggested by our data without item 4 (Table 3).

**Table 3:** Content of the subscales

**Total score**	
**Subscale**	**Summed questions**
**MORS warmth**	M 1+3+4+6+8+11+13
**MORS invasiveness**	M 2+5+7+9+10+12+14
**C-PRS closeness**	P 1+3+5+6+7+9+15
**C-PRS conflict**	P 2+4+8+10+11+12+13+14
**C-PRS conflict without P4**	P 2+8+10+11+12+13+14

#### Internal consistency

Table 4 shows the internal consistency by age of child and for all ages for the factors from both scales.

**Table 4:** Cronbach’s alpha for MORS (Child) and C-PRS sub-scales

**Age and number**	**Cronbach’s alpha**				
	**C-PRS**	**C-PRS**	**C-PRS**	**MORS (Child)**	**MORS (Child)**
	**closeness**	**conflict**	**conflict-4**	**warmth**	**invasiveness**
**2 (n=59)**	0.753	0.803	0.831	0.798	0.865
**3 (n=50)**	0.706	0.622	0.599	0.720	0.751
**4 (n=57)**	0.661	0.839	0.857	0.606	0.803
**All ages (n=166)**	0.719	0.787	0.804	0.731	0.814

Alphas were at or above the desirable level of 0.7 for all subscales. For C-PRS Conflict, the alpha rose when the subscale was constructed without item 4, suggesting that in this age range the scale is more robust without this item. Within age bands, alphas dropped below 0.7 for two evaluations for the C-PRS and one for the MORS (Child).

#### Criterion validity

**Figure 3:**
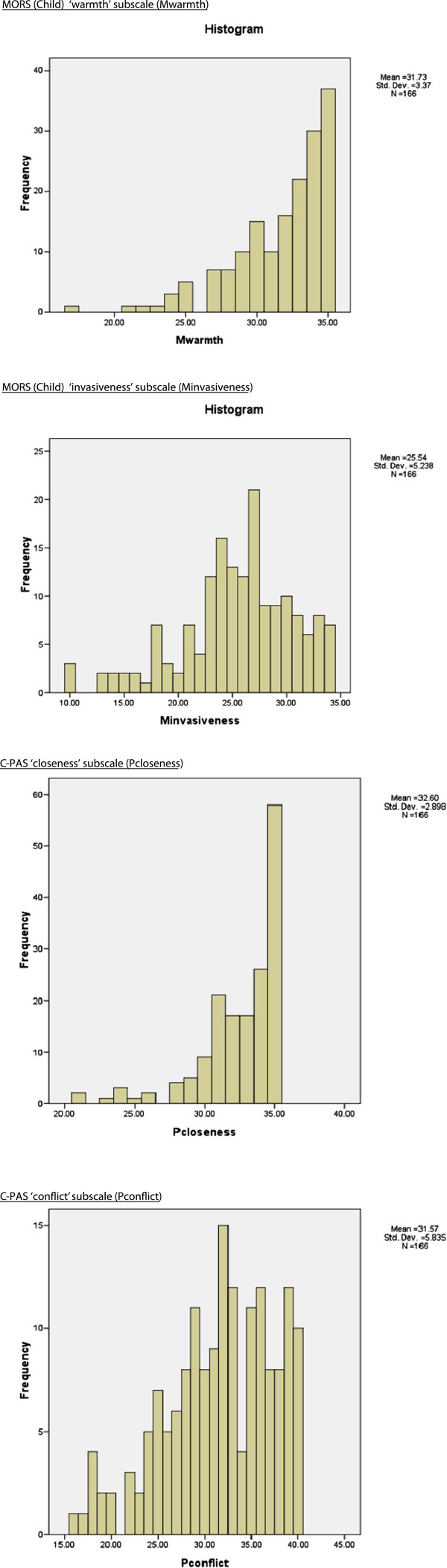
Score distribution for MORS (Child) and Child Parent Relationship Scales.

Figure 3 shows the histograms for the MORS (Child) invasiveness subscale and the C-PRS conflict subscale that are used to assess whether the scores approximately have a normal distribution. Both the MORS (Child) warmth subscale and the C-PRS closeness subscale were highly negatively skewed. Due to the non-normal distributions of these two subscales, and to allow for comparison of associations, correlations between all four subscale scores were tested by calculating the Spearman rank correlation coefficient (Table 5).

**Table 5:** Spearman correlations between subscale scores on MORS (Child) and the C-PRS

**Variable**	**MORS warmth**	**MORS invasiveness**	**C-PRS closeness**	**C-PRS conflict**	**C-PRS conflict (excluding item 4)**
**MORS warmth**	_	0.356*	0.439*	0.354*	0.363*
**MORS invasiveness**		_	0.346*	0.596*	0.601*
**C-PRS closeness**			_	0.376*	0.374*
**C-PRS conflict**				_	0.991*
**C-PRS conflict (excluding item 4)**					_

*Significant correlation at 0.01 level (2-tailed).

As expected, opposing constructs (e.g. warmth and conflict) correlate less than matching constructs (e.g. warmth and closeness). There was a stronger correlation between the MORS invasiveness and C-PRS conflict sub-scales than between the MORS warmth and C-PRS closeness sub-scales. The correlation was stronger when the C-PRS scale was calculated without item 4.

### External validation

When MORS (Child) data from the Family Links Nurturing Programme (FLNP) RCT was factor analysed together with the HOME inventory, two composite variables “Negative parenting” and “Supportive Parenting” were resolved (Table 6). Factor loadings for both the MORS (Child) and the HOME inventory items in these analyses were almost identical to those published in a similar analysis for the Sure Start evaluation using the C-PRS [20].

**Table 6:** Comparing factor loadings in sure start with C-PRS and FLNP RCT with MORS (Child)

**Variable**	**Negative parenting**		**Supportive parenting**	
	**Sure start**	**FLNP**	**Sure start**	**FLNP**
Invasion/Conflict	0.80	0.710	−0.14	−0.154
Warmth/closeness	−0.53	−0.590	0.39	0.155
Responsivity	−0.06	0.018	0.8	0.837
Acceptance	−0.09	−0.036	0.69	0.761
Harsh Discipline	0.70	0.789	0.20	0.150
Home chaos	0.60	0.672	0.24	0.057
				
Eigenvalue^a^	2.07	2.21	1.11	1.10

^a^In multivariate statistics, eigenvalues give the variance of a linear function of the variables. Eigenvalues measure the amount of the variation explained by each principal component (PC) and will be largest for the first PC and smaller for the subsequent PCs. An eigenvalue greater than 1 indicates that PCs account for more variance than accounted by one of the original variables in standardized data.

Table 6 compares the factor loadings for C-PRS and HOME from the SureStart evaluation with the factor loadings obtained in the MORS (Child) HOME factor analysis in the FLNP RCT. The factor loadings and the Eigenvalues are almost identical suggesting that MORS (Child) in 2–4 year olds performs in a way which is comparable to the C-PRS in 3 year olds when combined with items from the HOME inventory.

## Discussion

Valid and reliable outcome measures are needed to assess the impact of interventions to improve parent–child relationship quality and there is an age gap in validated measures in the pre-school years. This paper evaluates the performance of a new measure of parent–child relationships in this age range, an adaptation of MORS-SF a measure developed for babies. It also validates the C-PRS in two year olds.

The MORS-SF instrument was developed for use in primary care practice, usually by health visitors, and in research, as a unique tool designed to assess the nature of a mother’s internal working model of her infant in the months following the birth. It has been used in a number of contexts in England, Hungary and Australia as a component in screening to identify concerns about the developing dyadic relationship, and to assign mother-infant dyads to a relevant care pathway [23]. The assessment of attachment quality between parent and child is commonly a central concern when determining the need for interventions to improve parent–child relationships, and in tracking change during and following the intervention, not only in the post-partum, but also through later years of childhood. Given that the parent’s internal working model of their child is a core component of the attachment relationship, the use of an instrument that taps into elements of this model is clearly of potential value for practitioners.

The English government is moving to outcomes based management of health services. As parenting, particularly parenting in the first three years, is seen as key to public health improvement a new indicator has been proposed to measure the quality of parent-infant relationships which promote secure attachment [29].

This validation suggests that the MORS would be a good candidate for such an indicator; we have demonstrated that the MORS (Child) is psychometrically sound in 2–4 year olds and that parents find acceptable and easy to complete. On the other hand, validation of the C-PRS presented some issues in this age group. One item relating to ‘child avoiding physical contact and affection’ did not factor as expected from validation in older age groups. This may be because the item means different things at different ages. It is much less common for a 2–3 year old to avoid physical contact than an older child. The C-PRS was marginally more difficult for our sample of parents to complete than the MORS (Child), with some parents indicating that some items were worded in a way which was not as simple as could be and that some items did not apply as the child was not old enough.

The negative skew in the MORS (Child) warmth subscale and the C-PRS closeness subscale scores is not unexpected since low scores on both these subscales represent a relative lack of warmth and affection from the parent towards the child, which one might expect to be relatively infrequent in a general population sample. However, it could represent a social desirability bias, where parents are reluctant to portray themselves in a bad light by reporting low levels of these obviously positive behaviours. The correlation between the comparable scales of the two measures was, however, high.

MORS-SF has its basis in attachment theory; it aims to provide an assessment on two key axes of mother’s internal working models of their infants. Working models are generally considered to have a high degree of stability over time, because they are established as an outcome of many successive experiences and serve to regulate a person’s expectations of and behaviour towards their attachment figures [30]. Since the parent-infant attachment relationship is established largely during the first 18 months of the infant’s life, it is to be expected that the internal working model that a mother forms of her infant’s thoughts and feeling towards her will by then have become relatively stable. Hence the axes of perceived ‘warmth’ and ‘invasion’ can be expected to have on-going validity, even if the mother’s perceptions on these axes modify somewhat. The data from the current study confirm this, showing a factor structure in MORS (Child) that is virtually identical with that of MORS-SF [24].

We took advantage of data already collected using the MORS (Child) in the setting of an RCT to assess its factor structure as part of a composite measure of parenting. We were able to compare this with the factor structure of the C-PRS in the same composite setting using data published on the evaluation of Sure Start. The similarity in factor weightings of the two measures used in this way provides some evidence of the external validity of the MORS (Child) and confidence that it appropriate to use the MORS (Child) in this way. Further investigation of external validity would be valuable.

On the basis of this data we can safely recommend the MORS (Child) for assessment of the quality of the parent–child relationship in children aged 2–4 years. Given the very similar factor loadings with the MORS-SF in infants, and the theoretical expectation that working models do not change greatly in this age range without intervention, it seems very likely that this scale would also be valid in one year olds. Relationship quality in this age group where both ‘baby’ and ‘child’ are appropriate may be measurable using either of the MORS scales.

Our findings suggest that in the under 5 age group the MORS (Child) is a more robust measure than the C-PRS. This is perhaps not surprising as the C-PRS was developed in the US with primary school age children. Further work needs to be undertaken to evaluate the performance of the MORS (Child) in children of one year of age and to assess performance in children over four years. The likelihood of continuity between the MORS (baby) and the MORS (Child) needs confirming or refuting. If the MORS (Child) is to be used as an outcome measure to evaluate interventions, it will also be important to demonstrate sensitivity to change.

One limitation of this study is the question of generalising from our sample, which was confined to two areas, north Warwickshire and south Wales.

## Conclusions

Adapting the MORS-SF for children aged 2–4 years old produces a scale to assess parent–child relationships that is easy to use and performs well psychometrically. Whilst in many respects performance was similar to the C-PRS, in several respects MORS (Child) outperformed the C-PRS suggesting that this is the measure of choice in children under 5yrs.

## Abbreviations

C-PRS: Child–parent Relationship Scale; FLNP: Family Links Nurturing Programme; MORS-SF: Mother Object Relations Scale – Short Form; MORS (Child): Mother Object Relations Scale – Child Form.

## Competing interests

The authors declare that they have no completing interests.

## Authors’ contributions

DS coordinated the study and drafted the manuscript. FM carried out all statistical analyses on the dataset and helped to draft the manuscript. EF collected data, coordinated fieldwork and carried out qualitative and statistical analyses. JO developed the original instrument and helped to draft the manuscript. PK conducted the statistical analyses from the RCT and Sure Start datasets and helped to draft the manuscript. SSB conceived of the study, coordinated the development of the revised instrument and helped to draft the manuscript. All authors read and approved the final manuscript.

## Authors’ information

DS –MbChB, BMedSci, MSc, PhD, FHEA, FRCPCH, FRCP (Ed); Associate Professor of Child Health FM – BSc, MSc, PhD; Associate Professor of Psychology EF – BSc; Medical Student JO – BTech(Psychol); Senior Lecturer in Developmental Psychology PK – BSc, MSc, PhD; Research Fellow in Medical Statistics SSB - BM BCh MA PhD FFPH FRCP FRCPCH; Professor of Public Health.
